# Elevated Lipoprotein(a)-Associated Coronary Artery Disease in a 45-Year-Old Male

**DOI:** 10.7759/cureus.81684

**Published:** 2025-04-04

**Authors:** Fatima Abeer, Aasim A Wani, Ali Ahraz

**Affiliations:** 1 Internal Medicine, Government Medical College, Srinagar, IND; 2 Chemical Engineering, National Institute of Technology, Srinagar, IND; 3 Medicine, University of Dhaka, Dhaka, BGD

**Keywords:** cardiology, health disparities and global health, internal medicine/ general medicine, interventional cardiology, lipidology, metabolic syndrome and cardiovascular risk, preventive cardiology, vascular medicine

## Abstract

Premature coronary artery disease (CAD) in younger adults often arises from underrecognized risk factors such as elevated lipoprotein(a) (Lp(a)), a genetically determined lipoprotein with atherogenic and prothrombotic properties. We report a 45-year-old male with untreated hypertension, prior ischemic stroke, and significant tobacco use, who presented with exertional angina. Laboratory evaluation showed mildly elevated low-density lipoprotein cholesterol (LDL-C; 142 mg/dL), borderline low high-density lipoprotein cholesterol (HDL-C; 38 mg/dL), and markedly elevated Lp(a) (180 mg/dL). Coronary angiography revealed a chronic total occlusion of the proximal left anterior descending (LAD) artery, 90% stenosis of the left circumflex (LCx) artery, and Rentrop grade 3 collateral flow from a codominant right coronary artery. Due to financial constraints, revascularization was deferred. The patient was managed with high-intensity statins, dual antiplatelet therapy, beta-blockers, angiotensin-converting enzyme (ACE) inhibitors, and lifestyle modification. Over follow-up, he showed marked symptomatic improvement, enhanced left ventricular ejection fraction, and partial reversal of diastolic dysfunction. This case highlights the importance of Lp(a) screening in premature CAD and demonstrates that intensive medical therapy can stabilize high-risk patients when revascularization is not feasible.

## Introduction

Coronary artery disease (CAD) continues to be the leading cause of global morbidity and mortality, typically presenting later in life following prolonged exposure to classic risk factors such as hypertension, smoking, dyslipidemia, and diabetes mellitus [1]. Premature CAD, defined as disease onset in individuals younger than 45 years, represents approximately 4-10% of CAD cases and poses unique clinical challenges due to atypical presentations, diagnostic delays, and an increased likelihood of severe complications [2]. Although familial hypercholesterolemia (FH) and related genetic disorders are established contributors to early-onset CAD, a significant proportion of younger patients do not meet the diagnostic criteria for these hereditary conditions, underscoring the need to identify additional, frequently overlooked risk factors such as elevated lipoprotein(a) (Lp(a)) [2,3].

Lp(a), a genetically determined low-density lipoprotein (LDL)-like lipoprotein particle, is increasingly recognized as an independent and potent risk factor for accelerated atherosclerosis and premature cardiovascular events, even in individuals with otherwise mild lipid abnormalities [4]. It is not routinely included in standard lipid panels or widely used cardiovascular risk calculators despite mounting evidence linking elevated levels with adverse outcomes. Importantly, Lp(a) levels are largely unaffected by diet or statin therapy, making it a distinct, treatment-resistant risk factor. Structurally, Lp(a) contains an additional apolipoprotein(a) moiety that shares homology with plasminogen, thus conferring prothrombotic properties by interfering with fibrinolysis, in addition to its atherogenic potential. Clinically significant elevations of Lp(a) (>50 mg/dL) are present in approximately 20-25% of the general population, though prevalence varies significantly by ethnicity, with higher levels often observed in South Asian and African populations.

Despite this substantial prevalence, routine Lp(a) screening remains underutilized in clinical practice due to factors such as cost constraints, limited awareness, and variability in national recommendations [1,5]. Based on current recommendations, Lp(a) should be measured at least once in a lifetime, particularly in individuals with premature CAD, a family history of early cardiovascular disease, or unexplained recurrent events, with a threshold of >50 mg/dL (~125 nmol/L) considered clinically significant for increased cardiovascular risk [5]. While routine genetic testing for Lp(a) is not universally recommended, measuring its serum concentration serves as a reliable surrogate for genetic risk. Variations in the LPA gene, including the number of kringle IV type 2 repeats, account for the majority of inter-individual differences in Lp(a) levels. Large-scale epidemiological studies have consistently shown that elevated Lp(a) is associated with a two- to four-fold increased risk of premature CAD, particularly in individuals with coexisting metabolic risk factors. Although no specific pharmacologic agent exclusively targets Lp(a) in current practice, novel therapies, such as proprotein convertase subtilisin/kexin type 9 (PCSK9) inhibitors, can reduce Lp(a) by 20-30%, and antisense oligonucleotides against apolipoprotein(a) have demonstrated reductions of up to 80% in early-phase clinical trials. These advancements align with updated European Society of Cardiology (ESC) and American College of Cardiology/ American Heart Association (ACC/AHA) guidelines, which increasingly advocate for personalized approaches that address elevated Lp(a) in the context of recurrent or early-onset cardiovascular disease.

Here, we describe a 45-year-old male smoker from a rural area of Pulwama, South Kashmir, who presented with typical anginal chest pain and vomiting lasting approximately one hour. Electrocardiography revealed ST-segment depression in leads II and aVF, and high-sensitivity troponin T levels were within normal limits at initial testing. Given the ischemic symptoms, dynamic ECG changes, and high-risk profile, the patient was clinically diagnosed with a non-ST-elevation myocardial infarction (NSTEMI). He had a prior ischemic stroke involving the left middle cerebral artery, suggestive of a large-vessel origin, which may also be linked to elevated Lp(a). He was diagnosed with severe multivessel CAD involving significant stenosis of the left anterior descending artery (LAD) and the left circumflex artery (LCx). Coronary angiography demonstrated robust Rentrop grade 3 collateral circulation arising from a co-dominant right coronary artery, supplying the compromised myocardial territories. Laboratory findings revealed markedly elevated Lp(a) in the context of other traditional risk factors, including untreated hypertension, dyslipidemia, and smoking.

Although percutaneous coronary intervention (PCI) and coronary artery bypass grafting (CABG) were both advised following coronary angiography, the patient and family declined these options due to financial constraints, despite the availability of services at a higher-level referral center. The patient's rural residence likely contributed to delayed diagnosis and limited access to specialized cardiology services, including lipid profiling and early Lp(a) testing. Healthcare infrastructure in such settings is often constrained, with reduced availability of lipid-lowering therapies, delayed referrals for angiography, and limited preventive cardiovascular care, further compounding the risk in genetically predisposed individuals.

Follow-up echocardiography revealed a marked improvement in both systolic and diastolic function. While E/e′ ratios were not available, partial reversal of diastolic dysfunction was inferred from improved E/A ratio and increased ejection fraction. This case report underscores the critical importance of early and routine Lp(a) screening in younger individuals presenting with severe CAD, especially in populations lacking overt hereditary lipid disorders [1]. Additionally, it highlights the dual role of collateral circulation, which is protective against infarction yet insufficient to eliminate symptomatic ischemia, in guiding clinical management, particularly where revascularization options may be limited by financial or infrastructural barriers [4].

## Case presentation

A 45-year-old male farmer from Pulwama, South Kashmir, presented to the outpatient department on January 22, 2017, with acute-onset crushing and pressure-like substernal chest pain radiating to the left arm, accompanied by nausea, vomiting, and profuse sweating lasting approximately one hour. He described exertion-induced chest discomfort consistent with Canadian Cardiovascular Society (CCS) Class III angina. His past medical history was notable for untreated hypertension diagnosed five years prior, which remained unmanaged due to limited healthcare access and medication cost-related nonadherence, and a large-vessel ischemic stroke involving the left middle cerebral artery eight years earlier, resulting in residual mild right-sided hemiparesis. Cardioembolic workup for that event was not completed, and subclinical atrial fibrillation could not be ruled out due to a lack of ambulatory monitoring or contrast-enhanced echocardiography. He reported chronic cigarette smoking (20 pack-years), with no bidi or hukka use, and had not been taking any antihypertensive or lipid-lowering therapy. He denied any family history of premature coronary artery disease. Laboratory evaluation would later reveal markedly elevated Lp(a) and additional metabolic risk factors, including prediabetes and hepatic steatosis, consistent with a broader metabolic syndrome phenotype.

Initial electrocardiography showed ST-segment depression in leads II and aVF, with no reciprocal ST elevation or posterior leads available to assess for posterior wall ischemia. High-sensitivity troponin T was elevated at 184 ng/L. Laboratory testing revealed LDL cholesterol of 142 mg/dL, HDL of 38 mg/dL, triglycerides of 160 mg/dL, and markedly elevated lipoprotein(a) at 180 mg/dL. This LDL level is significantly above the recommended ESC 2019 secondary prevention target of <55 mg/dL for high-risk individuals. Transaminases were notably elevated (aspartate transaminase (AST) 367 U/L, alanine aminotransferase (ALT) 81 U/L), likely reflecting hepatic steatosis in the setting of metabolic syndrome. Fibrosis assessment via Fibrosis-4 Index for Liver Fibrosis (FIB-4) or elastography was not performed due to financial limitations. Mild anemia (Hb 11.5-13 g/dL) and metabolic acidosis (pH 7.33, pCO₂ 47 mmHg) were noted, interpreted as ischemia-related rather than indicative of compensated respiratory failure in the absence of chronic pulmonary disease indicators. Echocardiography showed preserved left ventricular ejection fraction (LVEF; 62%) and evidence of grade I diastolic dysfunction, though left atrial volume index was not recorded due to limited imaging parameters.

On initial physical examination, his vital signs revealed elevated blood pressure (150/90 mmHg), heart rate of 79 bpm, oxygen saturation of 99% on room air, and a BMI of 28.5 kg/m², indicating overweight status. Neurological examination confirmed mild residual right-sided weakness, while cardiovascular and pulmonary examinations were unremarkable, with no murmurs or adventitious lung sounds. Initial laboratory investigations on January 28, 2017, showed mixed dyslipidemia (Table 1). LDL cholesterol was mildly elevated at 142 mg/dL, high-density lipoprotein (HDL) cholesterol was reduced at 38 mg/dL, and triglycerides were within normal limits at 160 mg/dL. Notably, Lp(a) levels were markedly elevated at 180 mg/dL, far exceeding the normal upper limit (<30 mg/dL), significantly increasing his risk for premature atherosclerosis. Liver function tests revealed marked transaminitis (AST 367 U/L, ALT 81 U/L), raising suspicion of non-alcoholic fatty liver disease, later corroborated by abdominal ultrasound findings consistent with hepatic steatosis. A complete blood count indicated mild anemia (Hb 11.5-13 g/dL) but normal platelet and leukocyte counts. Arterial blood gases demonstrated mild metabolic acidosis (pH 7.33), mild hypoxemia (pO₂ 34 mmHg), and mild hypercapnia (pCO₂ 47 mmHg), suggestive of underlying metabolic and respiratory compromise, possibly related to cardiac ischemia or impaired perfusion (Table 2).

**Table 1:** Biochemical and metabolic panel The panel was done on January 28, 2017, and it includes liver, renal, lipid, and electrolyte markers with reference ranges. KFT - kidney function test; LFT - liver function test; LDL - low-density lipoprotein; HDL - high-density lipoprotein, AST - aspartate aminotransferase; ALT - alanine aminotransferase

Category	Assay	Result	Units	Reference range
Glucose metabolism	Glucose	106	mg/dL	60–100
Renal function (KFT)	Urea	22	mg/dL	10–50
Renal function (KFT)	Creatinine	1.08	mg/dL	0.50–1.50
Liver function (LFT)	Total bilirubin	1.2	mg/dL	0.2–1.2
Liver function (LFT)	Total protein	7.2	g/dL	6.2–8.8
Liver function (LFT)	Albumin	4.5	g/dL	3.5–5.2
Liver function (LFT)	AST	367	U/L	5–38
Liver function (LFT)	ALT	81	U/L	0–42
Lipid profile	Total cholesterol	204	mg/dL	50–200
Lipid profile	Triglycerides	72	mg/dL	0–170
Lipid profile	HDL	38	mg/dL	40–60
Lipid profile	LDL	141.6	mg/dL	10.0–135.0
Electrolytes	Calcium	9.29	mg/dL	8.40–10.20
Electrolytes	Phosphorus	2.09	mg/dL	2.30–4.70
Electrolytes	Sodium	138	mmol/L	136–150
Electrolytes	Potassium	4.4	mmol/L	3.5–5.1

**Table 2:** Summary of the patient's hematological, electrolyte, and arterial blood gas parameters, with corresponding reference ranges to support clinical interpretation

Category	Parameter	Baseline test	Follow-up test	Units	Reference range
Complete blood count	Hemoglobin (Hgb)	11.5	13	g/dL	M: 13.5–17.5, F: 12.0–15.0
Complete blood count	Total Leukocyte Count (TLC)	9.8	9.1	×10⁹/L	4.0–11.0
Complete blood count	Platelet Count (PLT)	212	208	×10⁹/L	150–450
Complete blood count	Mean Corpuscular Volume (MCV)	84	84	fL	80–96
Complete blood count	Mean Corpuscular Hemoglobin (MCH)	26	26.5	pg	27–33
Complete blood count	Neutrophils (N)	84	84	%	40–70
Complete blood count	Lymphocytes (L)	10	9	%	20–45
Electrolytes	Sodium (Na⁺)	150	134	mmol/L	136–145
Electrolytes	Potassium (K⁺)	3.8	4	mmol/L	3.5–5.1
Electrolytes	Calcium (Ca²⁺)	9.4	N/A	mg/dL	8.4–10.2
Arterial blood gas	pH	7.33	N/A	Unitless	7.35–7.45
Arterial blood gas	Partial Pressure of Oxygen (pO₂)	34	N/A	mmHg	75–100
Arterial blood gas	Partial Pressure of CO₂ (pCO₂)	47	N/A	mmHg	35–45
Arterial blood gas	Bicarbonate (HCO₃⁻)	24.3	N/A	mmol/L	22–26

Electrocardiography at presentation revealed ST-segment depression in inferior leads (II, aVF), consistent with myocardial ischemia without infarction, corroborated by normal high-sensitivity troponin T levels (Table 3). Echocardiographic evaluation on January 24, 2017, demonstrated mild left ventricular dilatation with preserved ejection fraction (EF) of 62%, mild anterior wall hypokinesia without other regional wall motion abnormalities, and evidence of grade I diastolic dysfunction (E/A ratio of 0.7, prolonged deceleration time of 283 ms), likely reflecting chronic hypertensive heart disease.

**Table 3:** Structural 2D echocardiographic measurements of cardiac chambers and walls obtained on February 9, 2017 LV - left ventricular; IVS - interventricular septum; LVPW - left ventricular posterior wall thickness; ES - end-systolic; ED - end-diastolic

Parameter group	Parameter	Value	Units	Reference range / comments
Aortic dimensions	Aortic root	30	mm	Normal: 25–37 mm
Atrial size	Left atrium	31	mm	Normal: <40 mm
Right ventricle	Right ventricle diameter	2.7	cm	Normal: <3.0 cm
LV dimensions	LV end-systolic diameter (ES)	30	mm	Normal: 20–40 mm
LV dimensions	LV end-diastolic diameter (ED)	49	mm	Normal: 37–56 mm
IV septum thickness	IVS thickness (ES)	14	mm	Normal: 6–11 mm (mild LVH)
IV septum thickness	IVS thickness (ED)	10	mm	Normal: 6–11 mm
LV posterior wall	LVPW thickness (ES)	14	mm	Normal: 6–11 mm (mild LVH)
LV posterior wall	LVPW thickness (ED)	10	mm	Normal: 6–11 mm
LV function	Ejection fraction	70%	%	Normal: 55–70%

**Figure 1:**
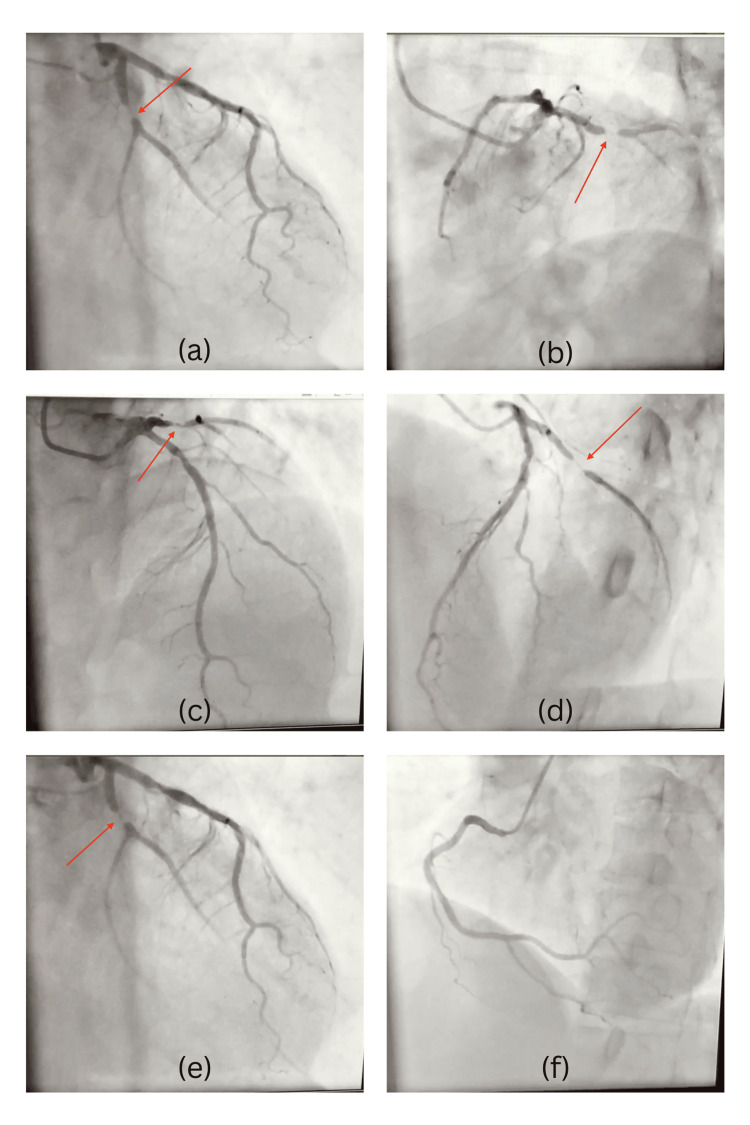
Coronary angiographic views demonstrating multivessel coronary artery disease (a) Caudal projection showing ~90% stenosis in the proximal left circumflex (LCx) artery just distal to the obtuse marginal (OM1) branch (arrow). (b) Cranial projection revealing chronic total occlusion of the proximal LAD artery immediately after the first septal (S1) branch. (c) Left anterior oblique view showing ~70% stenosis in the LAD distal to S1, with diminished distal opacification. (d) Alternate projection confirming the proximal left anterior descending artery (LAD) lesion, with luminal irregularity and impaired antegrade flow. (e) Close-up caudal view of the same LAD narrowing (c–d), further delineating the chronic nature of the lesion. (f) Right coronary artery (RCA) angiogram showing a co-dominant, patent RCA without stenosis, supplying Rentrop grade 3 collaterals to the LAD and LCx territories.

Given the patient's persistent symptoms and high-risk profile, invasive coronary angiography was performed on January 31, 2017, via the right radial artery approach (Table 4). This revealed severe multivessel coronary artery disease characterized by a 70% stenosis of the left anterior descending (LAD) artery immediately distal to the first septal branch (S1), and a critical 90% stenosis of the left circumflex artery (LCx) distal to the first obtuse marginal branch (OM1; Figure 1). Notably, a co-dominant right coronary artery (RCA) was angiographically normal but provided substantial collateral circulation (Rentrop grade 3, defined as complete filling of the distal vessel via collaterals), effectively supplying the myocardial territories compromised by LAD and LCx stenoses. The robust collateral formation likely prevented myocardial infarction, despite chronic significant stenosis.

**Table 4:** Doppler-derived functional parameters and qualitative assessments from the echocardiogram on February 9, 2017 LV - left ventricular; RWMA - regional wall motion abnormalities

Parameter group	Parameter	Value	Units	Interpretation / comment
Diastolic function	E wave velocity	0.6	m/s	Slightly reduced
Diastolic function	A wave velocity	0.8	m/s	E/A <1 suggests impaired relaxation
Diastolic function	Deceleration time	283	ms	Slightly prolonged
LV global function	LV function	Normal	N/A	Normal systolic motion
LV global function	Contraction	Normal	N/A	N/A
Regional wall motion	RWMA	Absent	N/A	No ischemia detected
Chamber assessment	Left atrium	Normal	N/A	N/A
Chamber assessment	Right ventricle	Normal	N/A	N/A
Chamber assessment	Right atrium	Normal	N/A	N/A

Upon admission, immediate management included loading doses of aspirin (300 mg), clopidogrel (300 mg), atorvastatin (40 mg), pantoprazole (40 mg), and beta-blockers to reduce myocardial oxygen demand. He received thrombolysis with streptokinase (1.5 million IU) due to initial suspicion of acute thrombotic occlusion alongside anticoagulation with enoxaparin. During hospitalization, medical therapy was optimized with continued beta-blocker therapy (metoprolol 25 mg twice daily), an angiotensin-converting enzyme (ACE) inhibitor (ramipril 2.5 mg daily), and high-intensity statins. At discharge, the patient was maintained on dual antiplatelet therapy (aspirin 75 mg daily and clopidogrel 75 mg daily), atorvastatin (20 mg twice daily), metoprolol (25 mg twice daily), and pantoprazole (40 mg daily). Financial constraints prevented the recommended revascularization procedures (PCI for LCx and CABG for LAD), prompting intensified medical management and rigorous lifestyle interventions, including smoking cessation and dietary modifications. At the three-month follow-up, the patient demonstrated substantial symptomatic improvement, reporting a reduction in angina frequency from approximately ten episodes to two episodes weekly. Echocardiographic reassessment on February 9, 2017, revealed normalization of anterior wall motion, improvement in diastolic function (E/A ratio improved to 0.9), and EF increased notably to 70%, underscoring the efficacy of comprehensive medical therapy and lifestyle modification in patients with severe coronary artery disease unable to undergo immediate invasive interventions.

## Discussion

This case underscores several essential facets of early-onset CAD, with particular emphasis on elevated Lp(a) as a dominant yet frequently overlooked factor. Traditionally, familial hypercholesterolemia or profound dyslipidemia are suspected in young patients presenting with severe atherosclerosis [1,2]. However, our patient's lipid profile revealed only mildly elevated LDL levels (142 mg/dL), modestly reduced HDL (38 mg/dL), and normal triglycerides (160 mg/dL). Far more notable was his markedly elevated Lp(a) of 180 mg/dL, significantly above the conventional risk threshold (<30 mg/dL). No family history of premature CAD had been reported, and the lack of Lp(a) screening prior to this event likely reflects limited clinical awareness, absence of a known familial pattern, and resource barriers in a rural healthcare setting. This discordance between LDL cholesterol and atherosclerotic severity strongly suggests that Lp(a) served as the principal atherogenic agent, reinforcing its importance in risk stratification when CAD occurs prematurely or without classic lipid abnormalities [6,7].

Structurally, Lp(a) closely resembles LDL but contains an apolipoprotein(a) moiety homologous to plasminogen, allowing it to inhibit fibrinolysis and promote thrombosis [3]. This dual atherogenic and prothrombotic action renders elevated Lp(a) particularly pathogenic in premature cardiovascular disease, notably in individuals with South Asian heritage or coexisting metabolic syndrome [4]. Conventional lipid-lowering agents, such as statins, exert minimal effect on Lp(a) and can sometimes further elevate it. Novel therapeutic avenues, including PCSK9 inhibitors and antisense oligonucleotides targeting apolipoprotein(a) (e.g., pelacarsen), as well as siRNA-based agents (e.g., inclisiran), show promise for targeted Lp(a) reduction [5,6]. However, high cost often limits their availability, especially in resource-constrained environments.

Coronary angiography disclosed severe multivessel disease, including a chronic total occlusion (CTO) of the proximal LAD with Rentrop grade 3 collaterals from a co-dominant RCA. These robust collaterals preserved myocardial perfusion sufficiently to avert infarction. Indeed, the patient's normal initial ejection fraction (62%) likely reflects sustained perfusion via collateral flow despite severe stenoses. Over follow-up, his ejection fraction (EF) increased to 70%, suggesting that myocardial stunning may have partially resolved, aided by improved microvascular perfusion and neurohormonal blockade-induced reverse remodeling. These Rentrop grade 3 collaterals maintained resting perfusion, yet could not fully prevent exertional ischemia, highlighting their dual implications, protecting against infarction but insufficient for exertional demands. Formal stress testing or cardiopulmonary exercise testing was considered but unavailable; symptom burden was used as a surrogate.

Beyond Lp(a), this patient displayed multiple features of incipient metabolic syndrome: borderline elevated BMI (28.5 kg/m²), hepatic steatosis with transaminitis (AST 367 U/L, ALT 81 U/L), and prediabetes (HbA1c 6.4%). Although full diagnostic criteria (e.g., waist circumference, fasting glucose) were not systematically documented, these findings strongly suggest metabolic dysfunction. Hepatic fibrosis (e.g., via FIB-4 or elastography) was not assessed due to resource limitations. Collectively, these metabolic derangements likely amplified systemic inflammation and endothelial dysfunction, compounding the prothrombotic milieu already driven by elevated Lp(a) [8,9]. Laboratory data showed mild anemia (Hb 11.5-13 g/dL) and mild metabolic acidosis with hypercapnia (pH 7.33, pCO₂ 47 mmHg). In the absence of chronic pulmonary disease indicators, we interpreted this as predominantly ischemia-related rather than chronic compensated respiratory failure. Tobacco use may have contributed further to overall hypoxic stress but did not appear to cause overt chronic respiratory insufficiency.

Although the presentation was consistent with non-ST-elevation myocardial infarction (NSTEMI), streptokinase was administered due to persistent ischemic chest pain, evolving ST depression, and lack of immediate PCI availability, an atypical but pragmatic decision in a resource-limited setting where clinicians may deviate from guideline-based care. Ultimately, financial constraints and patient preference precluded revascularization via PCI or CABG, including advanced CTO-specific interventions such as retrograde collateral PCI. Nonetheless, diagnostic angiography provided crucial anatomic and prognostic clarity, enabling physicians to tailor guideline-directed medical therapy (GDMT). Remarkable symptomatic relief, including a reduction in angina frequency from ten episodes per week to two, testifies to the efficacy of high-intensity statins, dual antiplatelet therapy, beta-blockers, and ACE inhibitors, supplemented by lifestyle modifications such as smoking cessation and dietary changes [10,11]. Alternative lower-cost strategies such as staged PCI or plain balloon angioplasty were discussed but ultimately deemed unfeasible due to cost and lack of structured follow-up access.

Since statins can paradoxically increase Lp(a) in certain patients, alternatives like ezetimibe or PCSK9 inhibitors were considered but deemed economically unfeasible. This case thus underscores the clinical relevance of universal Lp(a) screening in younger adults with severe or unexplained CAD, as endorsed by recent ESC and ACC/AHA guidelines, and the need for cost-effective, targeted screening strategies in low-resource areas. Adherence monitoring took the form of scheduled outpatient visits and phone consultations, given the lack of a formal cardiac rehabilitation program in the patient's rural locale. Similarly, long-term Lp(a) measurements were not planned, reflecting persistent financial and infrastructural barriers. In summary, this case illustrates how extreme Lp(a) elevation, in conjunction with hypertension, smoking, insulin resistance, and suboptimal healthcare access, precipitated aggressive premature CAD in a relatively young individual. Meticulous GDMT stabilized his clinical status, demonstrating the potency of a multifactorial intervention strategy, even without advanced revascularization [12-15]. As global cardiovascular guidelines increasingly recognize Lp(a) as a distinct therapeutic target, timely detection and specific interventions, whether pharmacologic or lifestyle-based, will become ever more pivotal to the management of premature CAD [16-18].

## Conclusions

This case highlights the multifactorial nature of premature coronary artery disease (CAD) in a 45-year-old man with markedly elevated lipoprotein(a), untreated hypertension, dyslipidemia, and a significant smoking history. Despite non-ST-elevation myocardial infarction (NSTEMI) and the absence of immediate percutaneous coronary intervention (PCI) options, diagnostic angiography revealed severe multivessel disease with robust collateral flow that prevented infarction yet did not eliminate ischemia. Intensive medical therapy, including high-intensity statins, dual antiplatelet therapy, and neurohormonal blockade, led to symptomatic and echocardiographic improvement, likely reflecting a combination of transient myocardial stunning reversal and early structural remodeling, though definitive revascularization was infeasible. Although multiple risk factors contributed to this patient's early-onset CAD, an extraordinarily high Lp(a) level (180 mg/dL) likely served as the major accelerant. The case underscores the importance of Lp(a) screening in younger adults with unexplained or severe disease, as well as the need for novel Lp(a)-lowering therapies. Further, it illustrates how socioeconomic barriers and limited access to advanced cardiac care can exacerbate disease progression in underserved populations. While the absence of extended follow-up and the retrospective nature of this report limit definitive outcome conclusions, the findings emphasize that comprehensive risk-factor management, coupled with early detection of elevated Lp(a), remains crucial for reducing premature cardiovascular morbidity.
